# A Case of Shoulder Presentation

**Published:** 1888-12

**Authors:** J. C. Beauchamp

**Affiliations:** Ga.


### A CASE OF SHOULDER PRESENTATION.

BY J. C. BEAUCHAMP, M. D., OF GA.

On January 30th, about 9 o'clock p. m., I was called to see a colored woman. She was 44 years old, weighed about two hundred pounds, and was the mother of fourteen children.

Found Mrs. W. present, one of those knowing midwives that are to be found in every community. From her I learned the negro woman had been in labor since 12 o'clock m. of the 29th. Upon examination I found a right shoulder presentation, with the hand and arm and six or eight inches of the umbilical cord protruding from the vulva. Pulsation had already ceased in the cord. I concluded to try to turn the child. I had read the modus operandi so often in the books, and heard it from lecturers, until I supposed it to be a very simple operation, very easy to be done. The waters had been discharged for 30 hours, the womb had contracted with powerful force, pressing the shoulder down in the peloic cavity, the os being clinched around the armpit like a vise.

After two hours of faithful effort to introduce the hand I failed. I then took off the arm at the shoulder joint, gave ½ grain morphine and made another attempt to turn. This time I finally managed to carry the hand into the womb, but the contractions were of such power that my hand would become paralyzed, and they were of such frequency, in fact almost continuous, that I did not recover muscular action sufficient to get the feet.

Being very much fatigued I sent for Dr. O., a neighboring practitioner, who reached the scene of operations about 7 o'clock a. m. on the 31st. Upon consultation we decided to put the patient under the influence of chloroform and make another attempt at version. This we did, but the anesthetic seemed to have no influeuce over the contractions and we failed to bring down the feet from the fact that the hand would become so deadened as to beperfectly useless. Dr. O suggested that we give a teaspoonful of tinct. opii and wait awhile for results, as he had read somewhere of spontaneous evolution under such circumstances. I accepted the suggestion and we gave 1 drachm tinct. opii at 9 o'clock a. m. Waited until 2 o'clock p m. and no change, we then gave her chloroform, and made another attempt at turning, but the hand would become so paralyzed that we could not reach the feet. At 3½ o'clock p. m. we gave her another teaspoonful of laudanum.

Saw her again at 9 o'clock p. m. in company with Dr. O. Examination did not show any change. We then gave her another teaspoonful tint. opii, ordered it repeated every five hours and went home.

I returned to see her at 9 a. m., February 1st. Dr. O. did not return. Found pulse rapid and weak, woman delirious at times, pains had about ceased, still I could not reach the feet; while there was no pain, still the womb was firmly contracted on the child. I saw that she was going to die, and that very soon, if something was not done.

I decided to perform evisceration, this I did with a blunt pointed bistoury, cutting the ribs in the right axillary region, removing the contents of the thorax, cutting back bone about the union of the dorsal and lumbar vertebræ, making traction by hooking my finger in walls of chest, the lower extremities were first delivered and then the head and chest about 10:30 a. m. This woman was in hard labor for 72 hours after I had seen her, and the midwife said she had been in labor 20 hours before I was called in. Your readers may say there is nothing in this case worth reporting, this is perhaps true, but at the same time it is a true statement, and it records a failure in performing an operation which is claimed to be simple. The remarkable thing about it is the woman got well

